# The lung microbiome in childhood-onset severe neuromuscular disease with respiratory insufficiency: rationale, current evidence, and opportunities for oxford nanopore long-read sequencing

**DOI:** 10.1186/s40348-026-00261-0

**Published:** 2026-09-19

**Authors:** Sebahattin Cirak, Vivien Grieshaber, Philip Müller, Cho-Ming Chao

**Affiliations:** 1 Division of Pediatric Neurology, Metabolics and Social Pediatrics, Department of Pediatrics and Adolescent Medicine, Ulm University Medical Center, Ulm University, Frauensteige 10, Ulm, 89075 Germany; 2 German Center for Child and Adolescent Health (DZKJ), Partner Site Ulm, Ulm, Germany; 3 Faculty of Health, Witten/Herdecke University, Witten, Germany; 4 Asklepios Children’s Hospital, Sankt Augustin, Germany; 5 Cardio-Pulmonary Institute (CPI), Universities of Giessen and Marburg Lung Center (UGMLC), Member of the German Center for Lung Research (DZL), Justus Liebig University Giessen, Giessen, Germany; 6 Louise Women’s and Children’s Hospital, Husener Straße 81, Paderborn, 33098 Germany

**Keywords:** Pediatric neuromuscular disease, Duchenne muscular dystrophy, Spinal muscular atrophy, Respiratory insufficiency, Lung microbiome, Dysbiosis, Oxford Nanopore, Long-read metagenomics, Antimicrobial resistance, Tracheostomy

## Abstract

In severe childhood-onset neuromuscular disease (NMD), ventilatory muscle weakness and ineffective airway clearance drive recurrent infections and chronic colonization that is often culture-negative, polymicrobial, or both. The lung microbiome framework offers a unifying model: altered microbial immigration, elimination, and growth can produce dysbiosis with pathobiont expansion and antimicrobial resistance (AMR). We propose that pediatric NMD may follow a distinct developmental trajectory in which early-life secretion stasis, viral insults, and frequent antibiotics perturb immune–microbiome crosstalk during lung growth, potentially “imprinting” long-term community structure. However, NMD-specific airway microbiome data remain sparse because most studies rely on culture or upper-airway sampling. Oxford Nanopore Technologies (ONT) long-read sequencing enables real-time metagenomics with AMR gene detection and can deliver same-day profiles (as short as ~ 6 h from sample to result in optimized workflows), but requires robust low-biomass controls and, in some settings, polishing or hybrid strategies to mitigate higher per-read error. A major limitation of metagenomic sequencing of respiratory samples is the high proportion of host DNA, bacterial reads may account for only about 1–5% of the total sequencing reads. We review microbiome principles relevant to pediatric NMD, summarize current evidence, and outline ONT-enabled study designs and translational priorities.

## Clinical context: why severe neuromuscular disease is a high-risk airway ecosystem

Severe neuromuscular disease (NMD) encompasses heterogeneous disorders of muscle, peripheral nerve, or the neuromuscular junction that converge on reduced inspiratory capacity, weak cough, and impaired airway protection. In pediatrics this phenotype is prominent in childhood-onset conditions such as Duchenne muscular dystrophy (DMD), spinal muscular atrophy (SMA), and congenital myopathies. Guidelines emphasize combined ventilatory pump and airway clearance failure, where clearance deficits can convert viral illnesses into bacterial pneumonia [1, 2]. Standard care therefore couples ventilatory support (typically NIV) with structured airway clearance and secretion management [2–4]. Because early-onset disease coincides with lung growth, some children never achieve normal peak cough flow or peak lung function, unlike adult-onset phenotypes; this developmental timing may shape airway microbial trajectories.Changes in the microbiota of the upper respiratory tract – e.g. due to factors such as diet, air quality, interpersonal interactions, antibiotic use -may be associated with dysbiosis of the lower respiratory tract. Additionally, the lower respiratory tract microbiome of children with severe NMD is subject to disease-specific factors.From a microbial ecology perspective, severe NMD imposes selection pressures through microaspiration, for example, in patients with bulbar dysfunction, gastroesophageal reflux, or swallowing incoordination, and reduced cough-mediated elimination, which may transiently or persistently influence the lung microbiome [3–5]. In children, these pressures act during lung growth and immune maturation and are compounded by recurrent viral illnesses, hospital exposures, and antibiotic courses. Airway instrumentation and antimicrobials further perturb niche conditions. These factors motivate rigorous, pediatric-focused characterization of airway microbial communities in NMD as potential mediators of infection susceptibility and targets for preventive strategies. We hypothesize that the upper airway acts as the principal immigration source for the lower airway, and that in severe NMD reduced clearance lowers microbial elimination while microaspiration raises immigration, so that the lower-airway community increasingly comes to resemble a dysbiotic upper-airway and oral source community enriched for pathobionts such as Pseudomonas, Haemophilus, Moraxella and Staphylococcus.

## Core principles of the lung microbiome relevant to neuromuscular respiratory failure

Sequencing studies indicate that the healthy lower airway is a low-biomass ecosystem whose signal often reflects the upper airway, consistent with ongoing microbial immigration and host-mediated elimination [6]. The lung microbiome is therefore best conceptualized as a balance between immigration, local growth conditions, and elimination by mucociliary transport and innate immunity [7–9]. In disease, dysbiosis often manifests as pathobiont overrepresentation and reduced community evenness rather than stable, high-diversity communities [8–10]. These principles are germane to NMD, where aspiration risk and impaired clearance can shift both immigration and elimination and where airway remodeling may favor pathogen expansion.

### The developing pediatric lung microbiome: why age, lung growth and the gut microbiome matter

Unlike adult cohorts, pediatric NMD intersects with postnatal lung growth and immune maturation, raising the possibility of “developmentally imprinted” dysbiosis [11, 12]. Early-life airway communities (roughly the first years after birth) are dynamic and influenced by antibiotics and respiratory viral infections, which can transiently destabilize communities and enable pathobiont blooms [11, 12]. In children with SMA or DMD, recurrent aspiration and cough failure may prolong epithelial exposure to microbial products during immune training, potentially biasing mucosal tolerance and inflammation [8, 9]. These considerations argue for age-matched controls, longitudinal sampling, and modeling of key clinical milestones (e.g., tracheostomy, initiation of assisted ventilation) rather than extrapolating adult patterns.

Across chronic respiratory diseases, culture-independent surveys commonly report shifts toward Proteobacteria (Pseudomonadota in current phylum nomenclature) including Pseudomonas, Haemophilus, and Moraxella, alongside reduced diversity and overrepresentation of oral taxa in the lower airways [9, 13]. Such patterns likely reflect changes in local oxygen tension, inflammation, nutrient availability, and antibiotic pressure. In children, these signatures can be episodic and temporally linked to viral infections (e.g. respiratory syncytial virus, influenza), which may transiently select for Proteobacteria-dominated states; repeated episodes in the setting of impaired clearance may bias community trajectories toward persistent pathobiont dominance including Moraxella, Haemophilus, Staphylococcus, Streptococcus, Neisseria, Prevotella and Corynebacterium species [11, 12].

In addition to these local processes, immune tolerance towards self-antigens and elimination of pathogens in the lungs are indirectly influenced by the gut microbiome (Gut-Lung Axis). It controls the pulmonary immune regulation essentially by three factors: 1. Production of short chain fatty acids, which influences expression of costimulatory surface molecules and antimicrobial peptides, phagocytosis of pathogens during infection, impair T-cell activation, and control of neutrophil migration; 2. Signaling through Pattern Recognition Receptors (PRR) of dendritic cells, which prime T-cells in local lymph nodes and stimulate their migration into different tissues to induce pathogen elimination by interleukin expression in alveolar macrophages and alveolar type 2 cells; 3. Segmented filamentous bacteria (SFB) interacting with immune cells and reducing lung colonization by germs such as MRSA and Aspergillus fumigatus [14]. The gut microbiome is known to be not only influenced by diet, but also by the mode of birth, feeding methods during infancy and exposure to antibiotics [15].

## What do we know specifically in neuromuscular disease?

Direct airway microbiome data in NMD remain limited and are largely culture-based, focusing on upper-airway swabs, which are limited by a potential overrepresentation of oral microbiome and an incomplete representation of the lower respiratory tract or tracheal aspirates. These approaches under-resolve polymicrobial structure, strain dynamics, and the AMR gene reservoir. In 77 patients with NMD, Stehling et al. detected potential pathogenic microorganisms in cough swabs in 51% (39/77) and found associations with lower forced vital capacity and cough peak flow [5]. In adults with long-term tracheostomy due to neuromuscular/neurologic disorders, Lepainteur et al. reported pathogenic bacteria in 90% (69/77) of tracheal aspirates, most commonly Pseudomonas aeruginosa (41%) and Staphylococcus aureus (44%); colonization was frequent even without infection, and empiric amoxicillin–clavulanate often lacked activity because of intrinsic resistance [16]. In tracheostomized children (median age 3 years), Severo et al. reported potentially pathogenic bacteria in 97.7% (43/44) of tracheal aspirates, dominated by P. aeruginosa (56.9%) with aminoglycoside and cefepime resistance linked to prior antibiotic exposure [17]. Together, these studies support a clearance-linked, AMR-pressured ecology but do not define distal-airway community composition or longitudinal, age-specific trajectories. Pediatric-focused sequencing studies with rigorous sampling and metadata are needed.

## Translational insight from mechanically ventilated populations

Because many patients with advanced NMD require long-term ventilation, ICU and mechanical-ventilation microbiome studies provide a relevant, though imperfect, analog. During mechanical ventilation, factors such as impaired airway clearance, altered respiratory dynamics, and exposure to antibiotics can alter microbial diversity. Most patients with NMD who require long-term ventilatory support are treated with noninvasive ventilation (NIV), while microbiome studies in the intensive care unit largely focus on invasive mechanical ventilation. Reviews report reduced airway diversity during ventilation with progressive dominance of opportunistic pathogens and, in some settings, enrichment of gut-associated taxa [18]. VAP frameworks increasingly view infection as an ecosystem transition shaped by antibiotics rather than a single exogenous pathogen [19]. This supports the hypothesis that in chronically ventilated NMD, dysbiosis may precede infection, as has been shown in other chronic lower-airway conditions such as cystic fibrosis, bronchiectasis and ventilator-associated pneumonia [19], and that longitudinal sampling could identify trajectories predictive of exacerbation risk.

## Why Oxford Nanopore long-read sequencing is particularly attractive

**Fig. 1:**
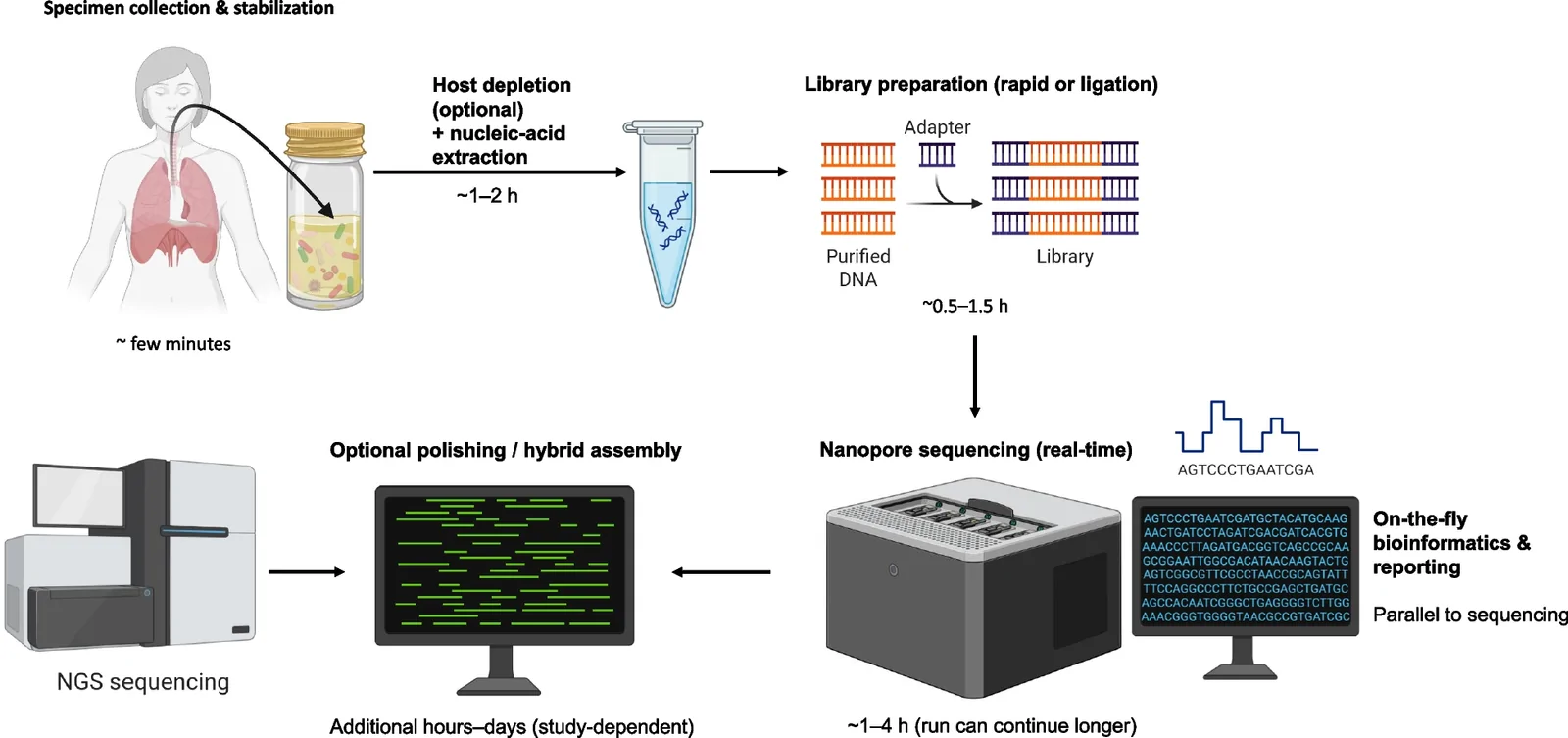
Specimen collection & stabilization

Most respiratory microbiome studies rely on short-read 16S amplicon sequencing, which summarizes community composition but offers limited strain resolution and little AMR context. ONT long-read sequencing enables real-time metagenomics and, with host depletion, can detect pathogens and AMR genes directly from respiratory specimens within a working day (as short as ~ 6 h from sample to result) [20, 21]. These findings are supported by a recent prospective clinical study comparing Illumina short-read and Oxford Nanopore long-read sequencing in bronchoalveolar lavage samples from 144 patients. Although Illumina showed higher sequencing accuracy and enabled more detailed microbiome characterization, ONT demonstrated reliable pathogen detection with a shorter turnaround time. Recent prospective studies support the clinical benefits of ONT-based diagnostics and demonstrate significantly improved detection of relevant respiratory pathogens including difficult-to-culture pathogens, across diverse clinical samples [22, 23]. These findings suggest that ONT may enable earlier microbiological decision-making and more timely antimicrobial optimization in respiratory infections.[24, 25]. Full-length 16S rRNA sequencing spanning V1–V9 can improve species-level classification [26]. In pediatric NMD, high host DNA fractions often necessitate optimized depletion or adaptive sampling [20, 27]. A key tradeoff is base accuracy: raw ONT reads have historically higher per-read error than short-read platforms (roughly 5–15% in earlier chemistries), though Q20 + workflows can exceed 99% single-read accuracy [28, 29]. For complex resistomes or strain tracking, polishing or hybrid strategies may be needed [28]. Table 1 and Fig. 1 summarize an illustrative same-day workflow. Recent clinical validations of nanopore and targeted-nanopore metagenomics in respiratory specimens, including head-to-head comparisons with short-read sequencing, support this approach [22–25, 30, 31].

**Table 1:** Illustrative same-day ONT metagenomic workflow (example total turnaround reported ~ 6 h in optimized protocols)

Workflow step	Purpose	Illustrative time range*	Notes for pediatric NMD/low biomass
Specimen collection & stabilization	Minimize degradation; document context	Minutes	Record timing relative to airway clearance, antibiotics, ventilation changes; rapid cooling/freezing when possible
Host depletion (optional) + nucleic-acid extraction	Reduce host DNA; enrich microbial signal	~ 1–2 h	Validate depletion for bias; include extraction blanks and mock communities
Library preparation (rapid or ligation)	Prepare DNA/RNA for sequencing	~ 0.5–1.5 h	Tradeoff between speed and yield; low-input protocols may be needed
Nanopore sequencing (real-time)	Generate reads until sufficient microbial depth	~ 1–4 h (run can continue longer)	Real-time classification can start immediately; depth needs depend on host fraction and community complexity
On-the-fly bioinformatics & reporting	Taxonomic profiling; AMR gene detection; QC	Parallel with sequencing	Use batch-aware contamination control; consider confirmatory qPCR/culture for clinical adjudication
Optional polishing/hybrid assembly	Improve base accuracy; resolve mobile elements/resistomes	Additional hours–days (study-dependent)	Most relevant for strain-level tracking and complex AMR contexts; may not be required for rapid pathogen detection

Annotation: *Time ranges are illustrative and vary by protocol, instrument, and bioinformatics; real-time analysis can provide interim results before run completion. ** ECFS CTN SOP Sputum induction for expectorating children and adults, V2.0

### Long-read versus short-read sequencing for the pediatric neuromuscular airway microbiome

We summarize here the main advantages and disadvantages of Oxford Nanopore long-read sequencing relative to high-throughput short-read sequencing for this setting, and Table 2 sets these out point by point. Short-read shotgun platforms provide very high depth and per-base accuracy across the bacteriome, virome, archaeome and mycobiome, and this depth is what makes them strong for quantitative diversity estimation and for detecting the rare biosphere, which makes up only a few percent of the lower-airway community but is informative for lung health [32]. In the airway, where bacterial reads may be only about 1 to 5 percent of all reads, short-read workflows commonly recover the microbial signal by sequencing very deeply, for example up to around 100-fold coverage on high-output instruments, by loading few samples per flow cell, and by removing host reads in silico through mapping against the human reference genome [20, 33]. Long reads improve species-level and sometimes strain-level assignment, in particular through full-length 16S sequencing across V1 to V9 [26], but the lower read depth per run and the low input loaded onto the flow cell mean that Oxford Nanopore is better suited to targeted, species-level pathogen questions than to high-throughput quantitative diversity. PacBio single molecule, real time sequencing offers a balanced approach with long reads at high base accuracy. Its technology is able to produce high-accuracy genomes through circular consensus sequencing, although at high cost and low throughput. A clear disadvantage to the Oxford Nanopore is the need for fragmentation, resulting in shorter reads (peak size 12kb) than Oxford Nanopore, making the reconstruction of highly repetitive regions more difficult. [34].

**Table 2:** Short-read (Illumina) versus long-read (Oxford Nanopore) sequencing for the pediatric neuromuscular airway microbiome

Feature	Illumina short-read	Oxford Nanopore (ONT) long-read
Sensitivity and specificity across kingdoms (bacteriome, virome, archaeome, mycobiome)	Very high depth and per-base accuracy. Shotgun covers all kingdoms well. Genus-level specificity is strong, and 16S species-level calls can be ambiguous [24]	Full-length 16S from V1 to V9 and long shotgun reads improve species and sometimes strain assignment, and direct RNA sequencing aids the virome. Confident species calls need high-accuracy basecalling [26, 35]
Required microbial biomass	Deep sequencing with in silico host removal handles a bacterial fraction of about 1 to 5 percent [20, 33]	Low-input kits and adaptive sampling enrich targets, but very low biomass magnifies contamination and host DNA [27]
Detection of the rare biosphere	High depth favours detection of low-abundance taxa at scale [32]	Places detected rare taxa more precisely, but lower throughput per run can undersample them. Adaptive sampling can target specific taxa [27]
False-positive and false-negative taxon assignment	Low per-base error, but chimeras and multi-mapping cause misassignment, and contamination drives false positives in low biomass [36, 37]	Long reads reduce ambiguous mapping and chimeras, but higher per-read error can cause false calls without high-accuracy basecalling [35]
Subclonal and low-frequency variants	High depth and low error make this a short-read strength [38]	Higher per-read error limits low-frequency variant calling, and this is improving with duplex and Q20 chemistry [28, 29]
Virulence and antimicrobial resistance determinants	Detects genes but fragments the resistome and loses genomic context [38]	Resolves full-length resistance operons, plasmid versus chromosomal location, and links to mobile elements [38]
Replication activity (growing versus dormant)	Coverage-based replication-rate estimation is better established with deep short-read data [39, 40]	Possible with adequate coverage. Neither platform separates dormant cells without RNA-based measures [39, 40]
Sequence and structural variants	Excellent for single-nucleotide and small variants, weaker for large structural variants and repeats [35]	Excellent for structural variants, repeats, phasing and genome assembly [35, 41]
Network analysis of polymicrobial communities	High sample throughput and depth support robust co-occurrence networks	Lower throughput per run limits large-scale networks, but long reads add molecule-level linkage [35]
Time from sampling to report	Batch-oriented, usually next day at the earliest [24]	Real-time, with interim results during the run and about 6 h in optimized protocols. Point-of-care friendly [20, 24]

*Comparison for the pediatric neuromuscular disease, low-biomass airway context. Platform performance evolves rapidly with chemistry and instrument version*

The clearest advantage of long reads for this setting is functional and structural resolution. Long reads span full antimicrobial resistance operons, link resistance genes to their genomic context, and separate chromosomal from plasmid-borne determinants, which matters because plasmid-borne genes are far more exposed to horizontal transfer through conjugation than chromosomal genes [38]. Long reads are similarly strong for structural variants, repeats and genome assembly [35], while short-read platforms retain the advantage for low-frequency and subclonal single-nucleotide variants because of their high depth and low per-base error [38]. Estimation of replication activity from coverage patterns is at present better established for deep short-read data, and neither platform separates dormant from actively growing cells without RNA-based measures [39, 40]. A recognized limitation of Oxford Nanopore is a historically higher per-read error, roughly 5 to 15 percent in earlier chemistries, although Q20 and R10.4 workflows now exceed 99 percent single-read accuracy and can yield near-finished genomes [28, 29, 41].

Where high per-read error affects individual genes of interest, precision can be recovered by combining the two approaches. By hybrid strategies we mean the use of short-read and long-read sequencing on the same sample, so that the accuracy and depth of short reads polish the long-read data and support quantitative diversity, while the contiguity of long reads resolves operons, mobile elements and structural variants [35, 38]. The main practical advantage of Oxford Nanopore is turnaround. Sequencing is real-time and can be read out during the run, with optimized protocols reporting results within about six hours from sample to result, whereas short-read platforms are batch-oriented and usually report the next day at the earliest [20, 24]. For the pediatric neuromuscular disease context this means that short-read sequencing remains preferable when the aim is deep quantitative diversity, rare-biosphere sensitivity or low-frequency variant detection, while Oxford Nanopore is particularly attractive for real-time pathogen and resistance profiling, for resolving mobile resistance elements, and for point-of-care turnaround in low-complexity samples with medium to high biomass [24].

## Methodological pitfalls and quality safeguards in low-biomass airway microbiome studies

Airway microbiome profiling is challenged by low biomass, upper-airway carryover, and extreme host-to-microbe DNA ratios, especially in pediatric sputum and aspirates. Depending on the sample type—such as sputum or bronchoalveolar lavage—bacterial reads may account for only about 1–5% of the total sequencing reads. Host depletion (e.g., differential lysis/nuclease protocols or adaptive sampling) can improve microbial yield but must be validated to avoid bias, since these steps can preferentially remove or retain particular taxa, for example through differences in cell-wall lysis, and should be checked against undepleted controls [20, 27]. Low-biomass specimens are vulnerable to reagent and laboratory contamination, making negative controls, mock communities, batch-aware design, and transparent decontamination reporting essential [36, 37]. ONT adds further nuances: while long reads improve taxonomic resolution and AMR gene context, higher per-read error than short-read platforms can complicate allele-level AMR calling and fine-grained strain tracking without high-accuracy basecalling and read-level QC/polishing [28, 29]. Standardizing collection (devices, timing relative to airway clearance, storage) and integrating clinical metadata remain prerequisites for interpretable microbiome–phenotype associations [2, 4, 16].

Specimen choice trades invasiveness for anatomical specificity. BAL best targets distal airways but is rarely feasible for routine longitudinal sampling in children with NMD. Tracheal aspirates and sputum are more scalable but are influenced by upper-airway carryover and device biofilms. Table 3 summarizes specimen options. Regardless of specimen, capture of ventilation mode, airway clearance regimens, aspiration risk markers, antibiotic windows, and infection adjudication criteria is essential for interpretable microbiome–phenotype associations [2, 4, 16].

**Table 3:** Specimen types for pediatric airway microbiome studies: strengths and limitations

Airway compartment	Specimen type	How obtained	Strengths	Key limitations and bias	Typical use in pediatric NMD studies
Upper airway	Nasal swab	Swab of the anterior nares or nasopharynx	Easy, low-risk and repeatable. Samples the nasal reservoir and immigration source	Poor proxy for distal airways and captures upper-airway flora	Noninvasive source-community measure and longitudinal surveillance
	Nasal lavage	Instillation and recovery of saline in the nasal cavity	Noninvasive with higher biomass than a dry swab	Upper airway only, with dilution effects and no distal surrogate	Comparator and source-community sampling across ages
	Oropharyngeal or cough swab	Swab of the oropharynx or a cough swab	Easy and low-risk. May approximate a source community for microaspiration	Overestimates oral taxa and is a poor distal proxy	Comparator, not a sole surrogate for the lower airway
	Exhaled breath (condensate)	Collection of exhaled air or condensate with a cooled collector	Fully noninvasive, feasible even in young or ventilated children	Very low biomass, high contamination risk and limited standardization	Emerging noninvasive option, needs strict low-biomass controls
Lower airway	Bronchoalveolar lavage (BAL)	Bronchoscopy with instillation and aspiration of saline	Closest approximation of the distal community, pairs with cytology	Invasive, limited feasibility, dilution and contamination risk	Mechanistic studies during clinically indicated bronchoscopy
	Protected brush or protected BAL	Bronchoscopy with a protected catheter or brush	Improves anatomical specificity and reduces upper-airway carryover	Invasive, limited availability and very low biomass	Selected research protocols when bronchoscopy is indicated
	Tracheal aspirate or endotracheal aspirate	Suction through a tracheostomy or endotracheal tube	Scalable and directly relevant to ventilated patients	Device biofilm contribution and upper-airway carryover. Colonization versus infection is hard to adjudicate	Longitudinal surveillance in ventilated or tracheostomy-dependent children
	Sputum (expectorated or induced)	Expectoration or induction, for example with hypertonic saline	Less invasive and repeatable, may reflect lower-airway processes	Strict adherence to Standard Operating Procedures, for example from ECFS**, is needed to limit upper-airway contamination	Feasible in milder NMD or older children with quality controls
Device	Device biofilm (tracheostomy tube, suction catheter, humidifier)	Sampling of a removed device or circuit component	Interrogates biofilm reservoirs and device-associated resistance genes	May not reflect the mucosal community, with timing and handling confounds	Adjunct sampling to link device care with persistence and resistance

For infants (0 to 1 year) and toddlers (2 to 3 years), noninvasive upper-airway specimens and, where a tracheostomy is present, tracheal aspirates are most appropriate. Induced or expectorated sputum becomes feasible mainly in older children (from about 4 to 6 years and above) who can cooperate. Bronchoalveolar lavage is generally limited to clinically indicated bronchoscopy at any age. **ECFS Clinical Trials Network SOP, Sputum induction for expectorating children and adults, V2.0

## Priorities for future research

Next-generation airway microbiome studies in childhood-onset NMD should be explicitly pediatric and translational. Longitudinal cohorts spanning key developmental windows are needed to define baseline states and test whether microbial shifts predict antibiotic-treated exacerbations, hospitalization, lung-function decline, or bronchiectasis. Beyond taxonomy, studies should quantify function (AMR burden and mobile elements) and track strains to distinguish persistence from repeated acquisition under frequent antibiotics. The functional capacity, in particular the resistome and the load of mobile elements, should be measured before and after antibiotic exposure. Longitudinal sampling is required to determine the treatment regimen and protocols. Pragmatic interventions embedded in care pathways (airway clearance escalation, aspiration mitigation, stewardship, tracheostomy care bundles) can test whether ecological endpoints are modifiable and clinically meaningful.

### Defining appropriate control populations and comparators

Truly healthy children rarely undergo bronchoscopy, so ‘healthy BAL’ controls are ethically challenging. Pragmatic options include opportunistic residual BAL or aspirates from children undergoing noninfectious airway evaluation, matched for age, sample type, and antibiotic exposure [12]. Disease controls with defined dysbiosis (e.g., CF or PCD) can support mechanistic comparison, and device-matched controls such as tracheostomized children without NMD can help disentangle device-associated biofilms [17]. Parallel oropharyngeal plus nasopharyngeal sampling can approximate the ‘immigration source’ community when distal sampling is limited, using pharyngeal (oropharyngeal and nasopharyngeal) swabs rather than oral or saliva sampling [12].

### From dysbiosis to intervention: testable clinical hypotheses

Defining an NMD-associated dysbiosis creates potentially treatable targets. If ONT identifies persistent pathogen dominance with a defined resistome, management could shift toward targeted strategies (e.g., inhaled antibiotics when appropriate) and away from broad empiricism. Strain tracking can distinguish biofilm persistence from reinfection, informing eradication versus suppression and device-care bundles. Ideally, studies should measure functional capacity before and after antibiotic exposure, focusing on the resistome and the load of mobile genetic elements. Longitudinal sampling should test whether function recovers after a single course of antibiotics. Continued follow-up should assess whether repeated treatment courses lead to a tipping point beyond which function no longer recovers. Long reads also enable exploration of adjunct approaches such as aerosolized bacteriophage therapy [42]. In chronically treated children, longitudinal metagenomics could quantify AMR reservoirs (including mobile elements) and provide objective markers for de-escalation and stewardship, as well as biomarkers to time escalation of airway clearance.

### Beyond the bacteriome: airway virome and mycobiome

Most NMD data emphasize bacteria, yet viruses and fungi shape airway ecology. Viral infections can perturb bacterial communities and precipitate pathobiont blooms [11, 12]. The airway mycobiome and bacteria–fungi interactions can influence inflammation and biofilm behavior [43]. Integrating bacteriome, virome, and mycobiome measurements will likely be important in tracheostomy-dependent children and will require optimized extraction (including RNA for viruses) and pipelines that accommodate mixed kingdoms while controlling low-biomass contamination.

### Disease-modifying therapies and shifting respiratory phenotypes

Disease-modifying therapies are reshaping childhood-onset NMD. In SMA, nusinersen, risdiplam, and onasemnogene abeparvovec improve survival and function, altering ventilation needs, aspiration risk, and healthcare exposure, all potential microbiome modifiers [44]. In DMD, exon-skipping and emerging gene/small-molecule therapies are changing trajectories, while steroids remain common and may influence infection risk and selection pressures [45]. Microbiome studies should document treatment era and therapy type and test whether microbiome features track with physiologic response (e.g., improved cough peak flow, reduced antibiotic burden) or therapy-related immunologic effects.

## Conclusion

Childhood-onset NMD with respiratory insufficiency is an understudied setting where altered mechanics, development, and intensive care exposures converge to shape the lung microbiome. Culture-based data already suggest frequent colonization and substantial AMR pressure in tracheostomy cohorts, motivating sequencing approaches that resolve polymicrobial communities and resistance reservoirs. ONT long-read metagenomics, paired with validated host depletion and rigorous low-biomass controls, can deliver same-day profiles and generate stewardship-relevant hypotheses. Key priorities include pediatric longitudinal cohorts, integration of bacterial, viral, and fungal components, and accounting for disease-modifying therapies that are reshaping respiratory phenotypes.

## Data Availability

No datasets were generated or analysed during the current study.

## Abbreviations

NMD: Neuromuscular disease
DMD: Duchenne muscular dystrophy
SMA: Spinal muscular atrophy
NIV: Noninvasive ventilation
BAL: Bronchoalveolar lavage
ETA: Endotracheal aspirate
VAP: Ventilator-associated pneumonia
CF: Cystic fibrosis
PCD: Primary ciliary dyskinesia
AMR: Antimicrobial resistance
ONT: Oxford Nanopore Technologies
